# Supplementary material for: Factors associated with pregnancy termination in six sub-Saharan African countries
Source: PLOS Glob Public Health. 2024 May 9;4(5):e0002280. doi: 10.1371/journal.pgph.0002280 (PMC11081391; doi:10.1371/journal.pgph.0002280)
Supplement: S1 Checklist — (DOCX) [file pgph.0002280.s001.docx]

STROBE Statement checklist of items of a study entitled Factors associated with Pregnancy Termination among women aged 15 to 29 in Sub-Saharan Africa

|  | Item No. | Recommendation | Page  No. | Relevant text from manuscript |
| --- | --- | --- | --- | --- |
| **Title and abstract** | 1 | (*a*) Indicate the study’s design with a commonly used term in the title or the abstract | 2 | A cross-sectional study design was employed |
|  |  | (*b*) Provide in the abstract an informative and balanced summary of what was done and what was found | 2 | The results showed that almost 6% of women aged 15-29 reported pregnancy termination with a higher prevalence in Tanzania (9%) and lowest in Ethiopia and Rwanda, 4.3% for each. Women with no education had 3.63 times higher odds of having pregnancy termination compared to women with higher education in Tanzania (AOR: 3.67 95 %CI 1.04, 12.90) while women with a primary and secondary level education were 1.78 times and 2.07 times as likely to terminate pregnancy in Ethiopia (AOR: 1.78 95% CI 1.12, 2.84) and Rwanda (AOR: 2.0795 %CI 1.01, 4.25) respectively. Women who were in richer household wealth quantile (AOR: 0.59, 95% CI0.39, 0.90) were less likely of having a pregnancy terminated compared to those in richest wealth quintile. In Tanzania, the likelihood of a pregnancy termination was associated with relationship to the household head; head (AOR: 3.66, 95% CI (2.32, 5.78, wife (AOR: 3.68, 95% CI 2.60, 5.12). |
| Introduction | | | |  |
| Background/rationale | 2 | Explain the scientific background and rationale for the investigation being reported | 4 | The prevalence of pregnancy termination is high in Africa, mostly underreported due to the law, cultural, religious and societal norms which often poses risks to the health and wellbeing of women therefore, making it an important public health issue. Hence, pregnancy termination continues to be a leading cause of maternal mortality among young women in Africa. |
| Objectives | 3 | State specific objectives, including any prespecified hypotheses | 5 | The aim of this research is to reappraise the level of pregnancy termination and associated factors among women aged 15 to 29 years in six sub-Saharan countries. |
| Methods | | | |  |
| Study design | 4 | Present key elements of study design early in the paper | 6 | Cross-sectional study design |
| Setting | 5 | Describe the setting, locations, and relevant dates, including periods of recruitment, exposure, follow-up, and data collection | 6 | Six countries with a DHS conducted not earlier than the year 2014 were selected: five from East Africa and one from West Africa. The selected countries were Nigeria (West Africa), Ethiopia, Tanzania, Kenya, Rwanda, and Burundi (East Africa). |
| Participants | 6 | (*a*) *Cohort study*—Give the eligibility criteria, and the sources and methods of selection of participants. Describe methods of follow-up  *Case-control study*—Give the eligibility criteria, and the sources and methods of case ascertainment and control selection. Give the rationale for the choice of cases and controls  *Cross-sectional study*—Give the eligibility criteria, and the sources and methods of selection of participants | 6 | Women aged 15-29 years included in the study from the six countries were 65,976 |
|  |  | (*b*) *Cohort study*—For matched studies, give matching criteria and number of exposed and unexposed  *Case-control study*—For matched studies, give matching criteria and the number of controls per case |  |  |
| Variables | 7 | Clearly define all outcomes, exposures, predictors, potential confounders, and effect modifiers. Give diagnostic criteria, if applicable 6 | 6 | Outcome variable: Pregnancy termination  Explanatory variables: Age measure as those within ages (15-29), residence (rural, urban), educational attainment (no education, primary, secondary, and higher education), occupation (professional/technical/managerial, clerical, sales, services, skilled manual, unskilled manual, agricultural and others), wealth index combined (poorest, poorer, middle, richer, and richest), sex of household head (male and female), relationship to household head (head, wife, daughter, daughter-in-law, granddaughter, sister, co-spouse, and other relative) and marital status (Never in union, currently in union/ living with a man, and formerly in union/ living with a man). |
| Data sources/ measurement | 8* | For each variable of interest, give sources of data and details of methods of assessment (measurement). Describe the comparability of assessment methods if there is more than one group | 6 | The Demography and Health Survey (DHS) data were retrieved from the measure online platform. The DHS uses three core questionnaires adapted from the MEASURE DHS project. These questionnaires include the household, women’s, and men’s questionnaires  The DHS recode (file IR) women dataset was used for the study. Recent DHS data on pregnancy termination in these selected countries were extracted from respective countries. Particularly data on women (aged 15–19) were extracted from all countries’ surveys. |
| Bias | 9 | Describe any efforts to address potential sources of bias | 6 | Probability proportional allocation to sample size was used |
| Study size | 10 | Explain how the study size was arrived at | 6 | The survey participants were selected using stratified and two-stage sampling methods: enumeration areas (EAs) in the first stage and households in the second stage. Each region in the selected countries was stratified into urban and rural areas. Then probability proportional allocation to sample size was made. Women aged 15-49 years from nationally selected households were then interviewed. |

Continued on next page

| Quantitative variables | 11 | Explain how quantitative variables were handled in the analyses. If applicable, describe which groupings were chosen and why | 7 | Frequencies and percentages for each country were computed to describe the demographic characteristics of respondents and outcome variables. |
| --- | --- | --- | --- | --- |
| Statistical methods | 12 | (*a*) Describe all statistical methods, including those used to control for confounding | 7 | Binary and multivariable regression analysis was performed |
|  |  | (*b*) Describe any methods used to examine subgroups and interactions |  |  |
|  |  | (*c*) Explain how missing data were addressed |  |  |
|  |  | (*d*) *Cohort study*—If applicable, explain how loss to follow-up was addressed  *Case-control study*—If applicable, explain how matching of cases and controls was addressed  *Cross-sectional study*—If applicable, describe analytical methods taking account of sampling strategy |  |  |
|  |  | (*e*) Describe any sensitivity analyses |  |  |
| Results | | | | |
| Participants | 13* | (a) Report numbers of individuals at each stage of study—eg numbers potentially eligible, examined for eligibility, confirmed eligible, included in the study, completing follow-up, and analyzed | 7 | 65,976 women aged 15-29 were included in the analysis. |
|  |  | (b) Give reasons for non-participation at each stage |  |  |
|  |  | (c) Consider use of a flow diagram |  |  |
| Descriptive data | 14* | (a) Give characteristics of study participants (eg demographic, clinical, social) and information on exposures and potential confounders | 7, 8 &9 | Socio-demographic characteristics |
|  |  | (b) Indicate number of participants with missing data for each variable of interest |  | No missing variable of interest |
|  |  | (c) *Cohort study*—Summarise follow-up time (eg, average and total amount) |  |  |
| Outcome data | 15* | *Cohort study*—Report numbers of outcome events or summary measures over time |  |  |
|  |  | *Case-control study—*Report numbers in each exposure category, or summary measures of exposure |  |  |
|  |  | *Cross-sectional study—*Report numbers of outcome events or summary measures | 10 | Prevalence of pregnancy termination |
| Main results | 16 | (*a*) Give unadjusted estimates and, if applicable, confounder-adjusted estimates and their precision (eg, 95% confidence interval). Make clear which confounders were adjusted for and why they were included | 12 &13 | Items under Marital status, household wealth quantile, relationship to the household head |
|  |  | (*b*) Report category boundaries when continuous variables were categorized | 8 | Age in five years classified based on DHS MEASURE |
|  |  | (*c*) If relevant, consider translating estimates of relative risk into absolute risk for a meaningful time period |  |  |

Continued on next page

| Other analyses | 17 | Report other analyses done—e.g analyses of subgroups and interactions, and sensitivity analyses |  | NA |
| --- | --- | --- | --- | --- |
| Discussion | | | | |
| Key results | 18 | Summarise key results with reference to study objectives | 15 | This study aimed to determine the prevalence of pregnancy termination and its associated factors among women in the age group of 15-29 in selected Sub-Saharan Africa countries. The current study showed that almost 6% of women aged 15-29 reported pregnancy termination with higher prevalence in Tanzania (9%) and lowest in Ethiopian and Rwanda 4.3% for each. The finding of this study was consistent with a multi-level study done in Nigeria, in Sub-Saharan countries, in Ethiopia, study done Nigeria which was 5%, 4.9%, 7.9%, 8.7% respectively |
| Limitations | 19 | Discuss limitations of the study, taking into account sources of potential bias or imprecision. Discuss both direction and magnitude of any potential bias | 17 | use of a cross-sectional |
| Interpretation | 20 | Give a cautious overall interpretation of results considering objectives, limitations, multiplicity of analyses, results from similar studies, and other relevant evidence | 17 | use of a cross-sectional study design that cannot describe cause and effect relationship of variables was the limitation of the study |
| Generalisability | 21 | Discuss the generalisability (external validity) of the study results | 6 | - A probability sampling technique was used.  - large sample size from different sub-Saharan countries was used in the study |
| Other information | |  | | |
| Funding | 22 | Give the source of funding and the role of the funders for the present study and, if applicable, for the original study on which the present article is based | 18 | No source of fund |

*Give information separately for cases and controls in case-control studies and, if applicable, for exposed and unexposed groups in cohort and cross-sectional studies.

**Note:** An Explanation and Elaboration article discusses each checklist item and gives methodological background and published examples of transparent reporting. The STROBE checklist is best used in conjunction with this article (freely available on the Web sites of PLoS Medicine at http://www.plosmedicine.org/, Annals of Internal Medicine at http://www.annals.org/, and Epidemiology at http://www.epidem.com/). Information on the STROBE Initiative is available at www.strobe-statement.org.
