# Supplementary material for: Factors associated with pregnancy termination in six sub-Saharan African countries
Source: PLOS Glob Public Health. 2024 May 9;4(5):e0002280. doi: 10.1371/journal.pgph.0002280 (PMC11081391; doi:10.1371/journal.pgph.0002280)
Supplement: Attachment — Submitted filename: Rebuttal letter for comment on pregnancy termination.docx [file pgph.0002280.s003.docx]

**Rebuttal letter**
1. We noticed you have some minor occurrences of overlapping text with the following previous publication(s), which needs to be addressed:

- <https://doi.org/10.17352/ijsrhc.000005>
- <https://hegelianizm.wordpress.com/2021/03/08/anti-abortion-laws-gender-based-violence-and-marginalization/>

In your revision ensure you cite all your sources (including your own works), and quote or rephrase any duplicated text outside the methods section. Further consideration is dependent on these concerns being addressed.

**Answer**

Rephrased and some other references are added to the introduction.

2. We have noticed that you have uploaded **Supporting Information files**, but you have not included a list of legends. Please add a full list of legends for your Supporting Information files after the references list.

**Answer**

The list of legends added after the reference list

3. In the online submission form, you indicated that "The Datasets used and/or analyzed during this study are available upon reasonable request from the corresponding author". All PLOS journals now require all data underlying the findings described in their manuscript to be freely available to other researchers, either 1. In a public repository, 2. Within the manuscript itself, or 3. Uploaded as supplementary information.
This policy applies to all data except where public deposition would breach compliance with the protocol approved by your research ethics board. If your data cannot be made publicly available for ethical or legal reasons (e.g., public availability would compromise patient privacy), please explain your reasons by return email and your exemption request will be escalated to the editor for approval. Your exemption request will be handled independently and will not hold up the peer review process, but will need to be resolved should your manuscript be accepted for publication. One of the Editorial team will then be in touch if there are any issues.

**Answer**

All data sets used/analyzed in this study and the ethics approval letters are attached or uploaded as supplementary information.

Additional Editor Comments (if provided):

There is need to pay extra attention to ensure that there are no missing words and the sentences are coherent. It may be a good idea to hire an editor to edit your manuscript.

**Answer**

Corrections made

**Comments to the Author**

Reviewer #1: I suggest that the authors **hire a copyeditor**.
The authors stated that the Datasets used and/or analyzed during this study are available upon reasonable request from the corresponding author. I suggest the author upload the data set as a supplementary file.

**Answer:** the data set (Weighted sample data) is uploaded

Abstract (p. 2)
Correct the total number of participants included in this study. The figure quoted is less than the one stated under the methods.

**Answer:** the total number of women included in the study has been changed from 65,415 to 74,652 after weighting the sampled data.

Introduction (p. 4,5)
Reference the second sentence of paragraph one. Referenced with reference number 2
Write the full meaning of WHO before using the abbreviation for the first time. Indicate if the figures in the last sentence of the first paragraph are worldwide figures. If yes, how different are these figures from the previous statistics?
I suggest you delete the first sentence of the third paragraph as it has no direct relation to the topic.
Mention the nations with 25% of unsafe abortions

**Answer:** updated worldwide data was included in the first paragraph

**Answer:** The full meaning of WHO has been written. We also updated the information and rephrased the concepts accordingly.

**Methods**
Population (p.6)
Write the full meaning of IR before using the abbreviation.

**Answer:** Individual Response

Justify why you chose women aged 15 to 29.

# Answer: Women aged 15 to 29 were chosen for this study because the age group 15-29 years has peculiar characteristics because they are young, vibrant, most sexually active, and spontaneous in decision-making and are still trying to figure out what the future holds in addition to the high child marriage rate in the study settings. Hence, this age group will most likely terminate an unplanned pregnancy on occurrence using crude and unsafe means (WHO, Adolescent Sexual and reproductive health, 2019).

# Results Sociodemographic characteristics (p. 7-9) Correct the narrative on the age of the respondents in Kenya. In Kenya, majority of participants were women aged 15 -19(35%) and 25 -29 (35%) respectively. Table 2: Recalculate the total sample for employment status for Ethiopia. The sample is more than n=9246 – corrected, it was because of the wrongly stated number for professional women Check the total sample for employment status, marital status, and Household wealth quantile for Kenya. The figures are not up to n=8257 corrected Check the total sample for marital status for Nigeria. It is more than n=22470.

# Answer: after running the weighted data for analysis almost all the figures has been changed.

# Prevalence (p.10) The total number of women aged 15 -29 who were included in this study is less than the figure stated.

# Answer: Corrected from 65,415 to 74,652 Include the overall prevalence of pregnancy termination in the study population. The overall prevalence of pregnancy termination in the current study was 6.25%

# Delete the last sentence under the prevalence since the figures are clearly stated in Table 2. It has been deleted.

# Factors associated with pregnancy termination (p.10,11) Include the results of the bivariate analysis in the narrative.

# Answer: the narratives have been Included in the revised version of the document. Correct the arrangement of the results on age and pregnancy termination for Burundi and Tanzania in the narrative; Burundi (AOR: 2.4 95%CI 1.5, 3.8), Tanzania (AOR: 2.7 95%CI 2.02, 3.57) on page 11 as women who were in the age group of 25-29 were more likely to have a higher chance of pregnancy termination compared to women aged 15-19 in Tanzania than Burundi.

# Answer: Corrected based on the weighted data

Women who were in the age group of 25-29 were more likely to have a higher chance of pregnancy termination compared to women aged 15-19 in ………. Tanzania (AOR: 2.75 95%CI 2.07, 3.65) followed by Burundi (AOR: 2.54 95%CI 1.58, 4.06) compared to women aged 15-19 in Tanzania than Burundi

**Discussion (p. 15-17)**
Clarify the fourth and fifth sentences of the first paragraph of the discussion;

This finding is lower compared to the study done among young women in another study that was done on the patterns of pregnancy termination

# Uganda, and in twenty-seven sub-Saharan countries which were 10.9%, 12%, and 16.5% respectively (36-38). The reason for this might be that the number of study participants in the former studies used a larger sample size which was DHS data from 50 countries.

# Answer

# It might be a grammatical error otherwise the study done on the patterns of pregnancy was done in another country apart from the mentioned countries in the document (Ethiopia). In short, the third study was done in Ethiopia on patterns of pregnancy termination which included 12 countries’ data sets.

# Also, revisit the explanation for the higher prevalence recorded on the fifth sentence since there was no prior mention of DHS data from 50 countries in the manuscript.

# Answer: Yes, but as stated in the reference numbers 44 to 46, the total number of DHS data used in the studies was 50. The study that was done in Ethiopia and Uganda used 12 & 1 data sets respectively whereas the study conducted in 27 sub-Saharan countries used 27 DHS data sets that gives a total number of 50 DHS datasets

# Discuss further the reasons for the higher rate of abortion in sub-Saharan Africa as compared to well-developed countries. Also, discuss studies that reported lower abortion rate among your study participants and explain the findings.

# Answer: Discussed in the discussion part on page 19 Discuss thoroughly why women with no education are more likely to terminate their pregnancies. P. 16.

# Answer: Discussed

# Conclusion (P. 17) Include the main contribution of the study to your field of practice or academic knowledge.

# Answer: some concepts have been added Strength and limitation (p. 17) Include limitations of DHS data set.

# Answer: Reporting and recall bias, particularly for age or other retrospective data relying on memory of a past event. PDF attachment (ethics) One of the PDF ethics attachments titled ʿMaternal Health Intervention in Reducing Maternal Mortality in Tanzania": is different from your research topicʿ clarify.

# Answer: We used a permission letter that was used for another research on “Maternal Health Intervention in Reducing Maternal Mortality in Tanzania” thinking that the title is related to the current topic which is on factors associated with pregnancy termination among women in Tanzania” but now we are granted a letter of permission for this specific study and we have attached it as supporting information.

Reviewer #2: Comment 1: Statistically this can't be claimed: Hence, with the rest of the included countries, the findings of this study can be generalized to the rest of the Sub-Saharan African countries.
Comment 2: Mention the version of DHS questionnaire for each survey and discuss the differences, if any. "The DHS uses three core questionnaires adapted from the MEASURE DHS project."---

- The Woman’s Questionnaire was used for each country included in this study.

Comment 3: How the sampling design was controlled during analysis? Mention in clearly with the we use the v005 sampling weight variable for bivariate as well as multi-variate analysis. "DHS collects data from household
samples using a two-phase stratified cluster design"

- Weights are used in Analysis to make sample data representative of the entire population so that the total sample size distribution looks like the country’s actual population distribution. Therefore, V005 (the women's individual sample weight was used to weigh the sampled data for each country included in this study. Since we used SPSS to run both bivariate and multivariate analyses, the following steps were used to compute.

1. COMPUTE WGT=V005/1,000,000 (a new variable was created which was used to weigh the sampled data
2. WEIGHT by WGT.
3. The weighted data were used to analyze the data.

Accordingly, the previously submitted figures in the document both in the descriptive and the inferential statistics have been changed in some cases.

Comment 4: Dependent variable - is this variable includes forced abortion as well? Please review.

**Answer:** Yes, any form of abortion including the forced one or pregnancy termination
Comment 5: Bi-variate analysis p-value must be set at 0.25 to include not only the statistically significant variables but to include the contextual important variable too. Read - Model Building chapter from Applied Logistic Regression book.

**Answer**: Included in the revision and narrations made to describe the bivariate analysis per the comment given.
Comment 6: The statistically significant independent variable in the bi-variate model must be checked for multicollinearity with variance inflation factor before taking them into the final model. The appropriate level of VIF for logistic regression must be used to rule out the confounding effect.

**Answer**: Multicollinearity between covariates was checked using the variance inflation factor (VIF) and VIF values greater than 10 indicate the existence of multicollinearity.
