# Supplementary material for: Factors associated with pregnancy termination in six sub-Saharan African countries
Source: PLOS Glob Public Health. 2024 May 9;4(5):e0002280. doi: 10.1371/journal.pgph.0002280 (PMC11081391; doi:10.1371/journal.pgph.0002280)
Supplement: Attachment — Submitted filename: Rebuttal letter 21.docx [file pgph.0002280.s004.docx]

**Rebuttal letter**

**Journal Requirements:**

1. Please review your reference list to ensure that it is complete and correct. If you have cited papers that have been retracted, please include the rationale for doing so in the manuscript text, or remove these references and replace them with relevant current references. Any changes to the reference list should be mentioned in the rebuttal letter that accompanies your revised manuscript. If you need to cite a retracted article, indicate the article’s retracted status in the References list and also include a citation and full reference for the retraction notice.

**Answer:** We have revised the reference list and some references also have been included per reviewers’ comment. We didn’t use retracted papers as far as our best knowledge.

2. We have noticed that you have a list of Supporting Information legends in your manuscript. However, there are no corresponding files uploaded to the submission. Please upload them as separate files with the item type 'Supporting Information'.

**Answer:** It is STROBE Statement—checklist of items that should be included in reports of observational studies which was attached as a legend. It had been also uploaded so far. Additionally, all data sets used/analysed in this study and the ethics approval letters had been attached or uploaded as supplementary information. Should I upload it again or should I upload the tables included in the manuscript too? (Kindly please)

**Answer to #Reviewer 1**

**Introduction**

Put a full stop at the end of the sentence; In Sub-Saharan Africa ………...preventable (6). **Answer:** full stop has been put
Put a full stop at the end of the sentence; Thirteen percent …………… unsafe abortion (13). **Answer:** full stop has been put
second sentence of the third paragraph. p. 3
Use a smaller letter to start the word ‟the” aim……. Last sentence of the last paragraph. p. 4. **Answer:** It has been corrected
**Methods**
These countries were selected because of the high prevalence of abortions in the regions where they are found in. I suggest you write it as; These countries were selected because of the high prevalence of abortions in the region” p. 4. **Answer:** It has been corrected
**Socio-demographic Characteristics**
Correct the sentence; In Kenya, Tanzania, Ethiopia, Burundi, Nigeria and Rwanda majority of the respondents were aged between 15-19. In Kenya majority 6100 (34.5) of the participants were between the ages of 25-29 not 15-19. The first sentence of the socio-demographic factors. p. 6. **Answer**: It has been corrected

**Factors associated with pregnancy termination**
Correct the sentence on the third paragraph: women living in rural areas compared to women living in rural. p. 10. **Answer:** It has been changed to…... compared to women living in urban areas…the figure that describes the odds ratio also edited.
Correct the sentence: women with a primary and secondary level are ............……... in Ethiopia. p. 14. The reported findings are for no education and primary education respectively, not primary and secondary education. **Answer:** It has been corrected.
Under marital status, correct the figure 72% (AOR: 0.28 95% 0.18, 0.41) for women in Nigeria who had never been in a union. The figure reported under table 3 was 5.35 (4.1, 06.98). p. 15,17. **Answer:** It has been corrected
Correct the results: Burundi, women who had relationship to the household ………............. in law 99% (AOR: 0.052, 95% CI 0.006, 0.48) less likely to terminate pregnancy. The 99% should be 95%. p. 15. **Answer**: it has been edited.
Also correct the word similarly on the last sentence of the result before table 3. p. 15. **Answer:** It has been corrected.

**Discussion**
Remove the almost in front of 6.25% on the second sentence of the first paragraph second. p. 19. The prevalence was 6.25%. **Answer:** edited
Further explain the statement; European countries are very developed with educated and civilized women who could forecast the consequences of pregnancy termination. Include reference as well. p. 19. **Answer**: Reference number 42 and 43 has been cited.
Include references for these sentences; Besides, the low socioeconomic status among women in the sub-Saharan countries might lead …………………… compared to the well-developed ones. p. 19. **Answer:** Reference number 44 to 47 has been added
Reference the sentences; The plausible explanation for this is that Ethiopia ………........... except for risky pregnancies. p. 19. **Answer:** Reference number 48 to 50 has been added and corrections made to the context of the statement.
Reference the sentence; Findings of this study ……………….................... because almost all women of this age group are sexually active. p. 20. **Answer:** Reference number 54 has been cited.
Reference this sentence; This may be related to the fact ………………….......... high salary/payment due to their low level of education. p. 20. **Answer**: reference number 56 has been cited.

Clarify the sentence; In almost 50% of countries included in this study, women with only primary education were more likely to undergo an abortion. p. 20. Are you referring to your current study or another study? Because in the current study you included only 6 countries. **Answer:** Yes, we are referring the current study and it is to mean that in the half (3 out of 6) of the study area(site) included in this study, women who attended only primary level education were more likely to terminate pregnancy.

Clarify the last part of the sentence on paragraph 4; maybe the programs may not include related courses for those who attended only primary education. What related courses are you referring to? p. 20. **Answer:** Most of the time reproductive and sexual health related topics are not included in the primary level education so that it will be difficult to have awareness regarding unplanned pregnancy, pregnancy termination and its complication among women who attended only primary education. Hence, we are referring to the reproductive and sexual health related courses.

Ethiopia just like most of the Sub-Saharan African countries has not legalized abortion (50). This is not true. In Ethiopia abortion is allowed by law based on certain conditions. p. 21…

**Answer:** Abortion law in Ethiopia----not fully legalized but allowed under a range of socioeconomic circumstances.

**Reference (**[Countries Where Abortion Is Illegal or Restricted | Time](https://time.com/6173229/countries-abortion-illegal-restrictions/)) and  **(**[Chart: The Legal Status of Abortion Worldwide | Statista](https://www.statista.com/chart/13680/the-legal-status-of-abortion-worldwide/))

The last paragraph of the discussion; join likelihood, it is one word. p. 21**.**

**Answer**: it has been corrected

**Answer to #Reviewer 2**

**Previous Comment 6:** The statistically significant independent variable in the bi-variate model must be checked for multicollinearity with variance inflation factor before taking them into the final model. The appropriate level of VIF for logistic regression must be used to rule out the confounding effect. You must use VIF cut-off of 2 for logistic regression as per the book I had recommended you earlier. This is very important as we don't want the confounding variable/s in the final multivariate model. So, kindly re-run the analysis and submit again. **Answer to this comment:** Multicollinearity between covariates was checked using the variance inflation factor (VIF) and VIF values greater than 10 indicate the existence of multicollinearity (This is ok for linear regression model only as it used Ordinary Least Square method so this is not correct for the logistic regression as it uses Maximum Likelihood method). **You must use VIF cut-off of 2 for logistic regression** as per the book I had recommended you earlier. This is very important as we don't want the confounding variable/s in the final multivariate model. So, kindly re-run the analysis and submit again.

**Answer:** Thank you so much for the comment. I learnt a lot.

Previously we only know that the cutoff point was 10 and that was why we responded that way even though the collinearity statistics showed the Variance inflation factor was less than 2 almost for all independent variables for all datasets included in this study. The other problem was that I couldn’t able to get the book you told me last time freely. Therefore, multicollinearity between covariates was checked using the variance inflation factor (VIF) and VIF values greater than 2 indicate the existence of multicollinearity. It was checked one by one for each country’s data set.

1. **Output for Burundi**

2. **Output for Ethiopia**

3. **Output for Nigeria**

4. **Output for Kenya**

5. **Output for Rwanda**

6. **Output for Tanzania**
