# Supplementary material for: Factors associated with pregnancy termination in six sub-Saharan African countries
Source: PLOS Glob Public Health. 2024 May 9;4(5):e0002280. doi: 10.1371/journal.pgph.0002280 (PMC11081391; doi:10.1371/journal.pgph.0002280)
Supplement: Attachment — Submitted filename: Plos Global paper on abortion.docx [file pgph.0002280.s005.docx]

**Reviewer Comments on the manuscript:**

Factors associated with pregnancy termination in Sub Saharan Africa

Rahel Nega Kassa, MSc. /PhD fellow, Emily Wanja Kaburu, MSc. /PhD fellow, Uduak Andrew-Bassey, MSc. /PhD fellow, Bonfils Nahayo, MSc. /PhD fellow, Ndayishimye Samuel, MSc. /PhD fellow, Saad Ahmed Abdiwali, MSc. /PhD fellow, Joshua Odunayo AKINYEMI, PhD

Plos Global Health

The authors have done a good job of addressing issues that were raised by the two reviewers, however the standard of English and the presentation of the purpose of the paper and the conclusion needs improving. The current approach is rather mechanical and does not portray a careful analysis and recognition of the social aspects of the problem. The writing needs to be improved such that the words used are appropriate and that terminologies are presented in a standard way. Using the example of comments made on the abstract and other comments provided below, the authors should review the whole paper and improve the writing to ensure that the sentences are comprehensible and intelligible. The authors should seek services of an independent editor to improve the presentation of the whole article and the sentences within it.

**Title**: Best to change to *Factors associated with pregnancy termination in Sub Saharan Africa*

*six Sub Saharan African countries*.

**Specific observations made on the abstract:**

- Where you mention Sub Saharan Africa, it should be The Sub Saharan Africa region or the region of Sub Saharan Africa.
- Remove the word *Therefore* at the beginning of the second sentence.
- Omit the words a cross sectional design was used in the third sentence and start from : *We use secondary data from the most recent ..........*
- The conclusion is not clear because the reference category is not mentioned.
- The conclusion only reflects on education but says nothing about other factors that emerged important in the analysis.

**Other comments**

- The word substantial is used to with respect to the 6% women that experienced abortion. Its not clear why 6% is considered substantial. Unless there is some good reason behind the use of that word e.g. compared to other regions or global estimates, 6% is pretty small. So I suggest the word substantial is not used.
- The findings point to poor socio-economic status as the key factor associated with abortion. I suggest that the conclusion should use this terminology because it summarises the list of factors that emerge as key; education, employment status, household wealth quintile and marital status (where being married uplifts women socio0economcically)
- Since the paper is abut the six Sub Saharan Africa countries, It should be stated that East Africa is part of the Sub Saharan Africa region.
- You need to start the discussion from the global perspective and end with the region in focus
  - Global (worldwide)
  - Africa
  - Sub Saharan Africa region
  - East Africa
  - Also be consistent , do not use the term Africa to mean SSA
