# Supplementary material for: Factors associated with pregnancy termination in six sub-Saharan African countries
Source: PLOS Glob Public Health. 2024 May 9;4(5):e0002280. doi: 10.1371/journal.pgph.0002280 (PMC11081391; doi:10.1371/journal.pgph.0002280)
Supplement: Attachment — Submitted filename: Rebuttal letter 21.docx [file pgph.0002280.s006.docx]

**Rebuttal letter**

The key issues of poor English, incomprehensible conclusions, and inconsistency in the use of the term Sub-Saharan Africa regions.

1. We have tried to correct the poor English
2. We made the conclusion clear
3. We used the term “Sub-Saharan Africa regions” consistently (started with capital letter when it comes to title and in first sentences otherwise used in a small letter).
