# Supplementary material for: Factors associated with pregnancy termination in six sub-Saharan African countries
Source: PLOS Glob Public Health. 2024 May 9;4(5):e0002280. doi: 10.1371/journal.pgph.0002280 (PMC11081391; doi:10.1371/journal.pgph.0002280)
Supplement: Attachment — Submitted filename: Rebuttal letter 21.docx [file pgph.0002280.s007.docx]

**Rebuttal letter**

**Journal Requirements:**

1. Please review your reference list to ensure that it is complete and correct. If you have cited papers that have been retracted, please include the rationale for doing so in the manuscript text, or remove these references and replace them with relevant current references. Any changes to the reference list should be mentioned in the rebuttal letter that accompanies your revised manuscript. If you need to cite a retracted article, indicate the article’s retracted status in the References list and also include a citation and full reference for the retraction notice.

**Answer:** thank you so much for the corrections

We have revised the reference list but we didn’t use retracted papers as far as our best knowledge. However, we corrected the following issues based on your valuable comment;

1. Reference number “3” was not mentioned in the previous manuscript.
2. Reference numbers 22 to 23 were wrongly cited in the statement included in the data source section. We have revised the articles and correctly inserted them to cite which justifies why the study enrolled only women aged 15 to 29.
3. Reference numbers 24 to 29 were cited to indicate the data sources for the six countries included in the study.
4. Reference numbers 55 & 56 were added during revision now in the discussion part
5. Finally, we recognized that there was an overlap in the citation process and we currently have 57 references which is less than the previous one which was 61.

2. We have noticed that you have cited Table 4 in the manuscript file but there are no corresponding tables in the manuscript. Please amend your manuscript to include this table, noting that tables should not be uploaded as individual files.

**Answer:** Thank you for the comment but I was not able to get Table 4 cited in the text. However, we added two tables; one (Table 4) for the bivariate analysis and the other(Table 5) to show the values of the variance inflation factor (VIF) in the revised manuscript.

3. We have noticed that you have uploaded Supporting Information files, but you have not included a list of legends. Please add a full list of legends for your Supporting Information files after the references list.

**Answer: okay, thanks for the comment**

**List of Legends**

STROBE Statement checklist of items of a study entitled Factors Associated with Pregnancy Termination among Women aged 15 to 29 in Sub-Saharan Africa

Table 1: Sociodemographic characteristics of women within the age group of 15 to 29 in six sub-Sahara African countries

Table 2: Prevalence of pregnancy termination among women aged 15-29 in six sub-Sahara African countries

Table 3: Bivariate analysis to show the association of sociodemographic variables and pregnancy termination among women aged 15 to 29 in six sub-Sahara African countries.

Table 4: Table that shows multicollinearity between covariates

Table 5: Factors associated with pregnancy termination among women aged 15 to 29 in six sub-Sahara African countries

**Review Comments to the Author**

Please use the space provided to explain your answers to the questions above. You may also include additional comments for the author, including concerns about dual publication, research ethics, or publication ethics. (Please upload your review as an attachment if it exceeds 20,000 characters)

Reviewer #1: The quality of the manuscript has improved substantially.

**Answer**: thank you for the positive feedback

Results
Sociodemographic
In Tanzania majority (36.9%) of the participants were rather working in the agriculture sector. p 6

**Answer:** corrected

Discussion
Rephrase the last sentence of paragraph one. p14

**Answer:** rephrased
Delete 'which were' after Norway in the first sentence of paragraph two. P 14

**Answer:** deleted
Reference the last but one sentence of paragraph five ‘ it can only be accessed in limited private clinics’ p 15

**Answer:** referenced with reference number ”55”
Also reference the last sentence of paragraph five ‘The richest women are more civilized, and career driven and may consider pregnancy termination/abortion due to their career pursuit’. p 15

**Answer:** referenced with reference number”56”

Reviewer #2: The authors have mentioned that they have used p-value of 0.25 in the bi-variate analysis as cut-off to include independent variables in the final model but this is not seen in any Tables presented not it is discussed in the result section. Further, they also mentioned in the method section that VIF cut-off of 2 was used to take variables with p-value less than 0.25 in the final model but the results/tables do not show it and even none of the text in the result section described it. Thus, it is recommended to add a table with bi-variate analysis results separately, like Table 3, in the manuscript and show the actual p-values there for all the countries. Table 3 or separate section must show/mention the VIF values for each country for each variables included in the final model so that the Table 3 can be taken as the final result and conclusions can also be taken as valid and reliable.

**Answer:** table 3 & table 4 are added to show the bivariate analysis and multicollinearity test using variance inflation (VIF) respectively. We also attached the output of SPSS that shows the VIF result as supporting information.

It is not clear what this statement means in the result section: "After running the significantly associated variables in multivariate logistic regression except for type of residence and sex of household, the rest variables were significantly associated with the outcome variable at least in one of the six countries."

**Answer:** we paraphrased it to make it clearer

**Reviewer Comments on the manuscript:**

Factors associated with pregnancy termination in Sub Saharan Africa

Rahel Nega Kassa, MSc. /PhD fellow, Emily Wanja Kaburu, MSc. /PhD fellow, Uduak Andrew-Bassey, MSc. /PhD fellow, Bonfils Nahayo, MSc. /PhD fellow, Ndayishimye Samuel, MSc. /PhD fellow, Saad Ahmed Abdiwali, MSc. /PhD fellow, Joshua Odunayo AKINYEMI, PhD

Plos Global Health

The authors have done a good job of addressing issues that were raised by the two reviewers, however the standard of English and the presentation of the purpose of the paper and the conclusion needs improving. The current approach is rather mechanical and does not portray a careful analysis and recognition of the social aspects of the problem. The writing needs to be improved such that the words used are appropriate and that terminologies are presented in a standard way. Using the example of comments made on the abstract and other comments provided below, the authors should review the whole paper and improve the writing to ensure that the sentences are comprehensible and intelligible. The authors should seek the services of an independent editor to improve the presentation of the whole article and the sentences within it.

**Title**: Best to change to *Factors associated with pregnancy termination in Sub-Saharan Africa*

*six Sub-Saharan African countries*.

**Specific observations made on the abstract:**

- Where you mention Sub Saharan Africa, it should be The Sub-Saharan Africa region or the region of Sub Saharan Africa.

**Answer**: all are replaced with “the Sub-Saharan Africa Region”

- Remove the word *Therefore* at the beginning of the second sentence.

**Answer:** the word “Therefore has been deleted”

- Omit the words a cross sectional design was used in the third sentence and start from : *We use secondary data from the most recent ..........*

**Answer:** corrected accordingly

- The conclusion is not clear because the reference category is not mentioned.

**Answer:** the reference category was included

- The conclusion only reflects on education but says nothing about other factors that emerged important in the analysis.

**Answer:** educational status was changed to socio-economic status because the socio-economic status of the woman includes educational status too in addition to the other significantly associated factors like marital status, employment status, and so on.

**Other comments**

- The word substantial is used to with respect to the 6% women that experienced abortion. It's not clear why 6% is considered substantial. Unless there is some good reason behind the use of that word e.g. compared to other regions or global estimates, 6% is pretty small. So I suggest the word substantial is not used.

**Answer:** we initially used the word substantial considering the risk of pregnancy termination especially when it comes to developing nations like in this study setting. Because 6% of the total population included in this study (74,652) which is 4,479 women had a history of pregnancy termination. However**,** after a revision of several articles related to pregnancy termination, and decided that the current study’s finding is less compared to the global findings and another study which was conducted in low- and middle-income countries. Therefore, we avoided the word ”substantial”

- The findings point to poor socio-economic status as the key factor associated with abortion. I suggest that the conclusion should use this terminology because it summarises the list of factors that emerge as key; education, employment status, household wealth quintile and marital status (where being married uplifts women socioeconomically)

**Answer:** corrected accordingly.

- Since the paper is about the six Sub Saharan Africa countries, it should be stated that East Africa is part of the Sub Saharan Africa region.

**Answer:** corrected in a way that East Africa is also part of SSA but we intended to justify why most (5 out of 6) of the included countries were from East Africa

- You need to start the discussion from the global perspective and end with the region in focus
  - Global (worldwide)
  - Africa
  - Sub-Saharan Africa region
  - East Africa
  - Also be consistent, do not use the term Africa to mean SSA

**Answer:** corrected per the comment
